# Editorial: Insights in auditory cognitive neuroscience: 2021

**DOI:** 10.3389/fnins.2023.1192459

**Published:** 2023-04-11

**Authors:** Marc Schönwiesner, Claude Alain

**Affiliations:** ^1^Institute of Biology, Faculty of Life Sciences, Leipzig University, Leipzig, Germany; ^2^Department of Psychology, Faculté des Arts et des Sciences, Université de Montréal, Montreal, QC, Canada; ^3^Department of Psychology, University of Toronto, Toronto, ON, Canada; ^4^Rotman Research Institute, Baycrest Hospital, Toronto, ON, Canada

**Keywords:** auditory, pitch, MMN (mismatch negativity), speech-in-noise, hemispheric asymmetries, voice, what/where system, hearing disorders

Imagine an expensive research and development meeting at a large company. The presenter: “We have our top people working on this. Our top people!” This is how we feel about the many recent breakthroughs in auditory cognitive neuroscience research. Researchers like Tim Griffiths, Robert Zatorre, Andrew Oxenham, and the other contributors to this Frontiers' Research Topic have shaped and advanced the field for years. This collection of ten short perspective papers aims to provide a readable overview of several current (and, in many cases, timeless) topics in auditory cognitive neuroscience through the vantage point of some of the main actors. The papers are best enjoyed as a collection rather than independently because of the many interconnections between the topics they discuss, some of which we will point out here.

We start with topic of processing and representation of critical auditory features. The mechanism of pitch perception is among the oldest such topics in hearing science, going back to Strutt (1907). The brain encoding of time-based pitch cues has seen strong empirical support using delay-and-add noise in brain imaging studies (Griffiths et al., 1998). A classical study by Oxenham et al. (2004) demonstrated that time-based cues are not sufficient and that pitch perception also requires correct cochlear frequency-to-place mapping of the spectral components of the stimulus. After over a 100 years of research, the relationship between these two cues in pitch perception and representation is still under debate. The perspective by Oxenham discusses recent developments and directions in the study of pitch coding and perception.

From pitch extraction is the extraction of voice features: Pascal Belin's discovery of the temporal voice area in 2001 (Belin et al., 2000) opened up new research into the cortical processing of voices and non-speech vocal sounds. This area around the middle of the superior temporal sulcus responds more strongly to voices than other sounds. There is some discussion of whether this area is processing speech rather than voice information, which is reminiscent of the debate around whether the fusiform face area genuinely represents faces or any stimuli that observers have acquired expertise with (Gauthier et al., 1999). Here Trapeau et al. present evidence-based arguments to support the role of the temporal voice area in genuine voice processing.

The mismatch negativity is one of the most popular neural metrics to study preattentive processing, predictive coding mechanisms, auditory memory, and many other phenomena. Its discovery in late 1978 by Finish psychologist Risto Nätäänen created a paradigm shift in auditory neuroscience. Tervaniemi discusses the development of stimulation paradigms from simple sine tones to complex multi-feature sounds and paradigms, including recent efforts to achieve ecological validity in experiments with such tightly controlled and repetitive stimuli. These new developments will ensure that the mismatch negativity remains among the most significant and versatile tools in auditory cognitive neuroscience for years to come.

Our understanding of the function and organization of the human primary (core) auditory cortex needs to catch up to that of the visual cortex. The auditory core is much smaller than V1 and is divided into subfields, nested on the superior temporal gyrus. Several functional and anatomical markers have been discovered and allow some non-invasive access, for example, increased myelination (Sigalovsky et al., 2006), the 40-Hz auditory steady-state response (Gutschalk et al., 1999), or a peak in the slope of the magneto-encephalographic response at about 20 ms (Lütkenhöner et al., 2003). Simon et al. argues that early time-locked high gamma band responses to natural speech can track primary cortical activity, adding a robust and ecologically valid method to study primary auditory cortex function non-invasively.

We now turn to the organization of the auditory system. Zatorre provides a perspective of hemispherical asymmetries in music and speech processing, in which his group has contributed significant theoretical and empirical advances. This is a topic with deep historical roots going back to the recognition of lateralized language areas by Broca and Wernicke in the late 19th century. Zatorre unifies recent results on the processing of musical pitch patterns in auditory networks of the right hemisphere (and complementary lateralization of speech sounds) in the framework of spectrotemporal modulation processing. The paper discusses the importance of low-level differential sensitivity to acoustical features of communication sounds (bottom-up) and high-level modulation of asymmetries by learning, attention, or other top-down factors.

A central concept of sensory processing in the cortex is that of partially segregated streams with different functions. This idea was initially conceived to explain different sensitivities, and latencies in cortical fields along the visual pathway (Schneider, 1969; Mishkin and Ungerleider, 1982; Goodale and Milner, 1992) and later applied to audition by Rauschecker and Tian (2000) with the proposal of “what” and “where” pathways. This idea was reconceptualized several times, and the dual pathways have lost their initial clear functional separation and are now often referred to by location. These ventral and dorsal processing streams originate in the secondary (belt) auditory cortex in rostral and caudal fields, which then connect to different downstream areas in the frontal and parietal cortex. A recurrent functional distinction that has held up since the original studies in non-human primates is that rostral fields tend to be more involved in sound recognition and caudal areas more in sound localization. Scott and Jasmin discuss the origins and recent developments of the dual stream concept and its interaction with speech and voice processing of simultaneous talkers.

The feedback or top-down auditory projections is another principle of brain organization with powerful implications. The cortico-fugal pathway, the thickest efferent projection in the human brain after the pyramidal tract, instructively illustrates this. McAlpine and de Hoz discuss how adaptation in such feedback pathways of the auditory system aids in adaptive en- and decoding of complex sounds by building a representation of their statistical structure at different time scales. Exploring these feedback loops at different granularities, from *in vivo* recording to human neuroimaging, may reveal the fundamental listening processes.

Finally, we turn to topics in more applied auditory neuroscience. Griffiths provides an overview of recent work in the lab on predicting speech-in-noise ability based on performance with non-speech material in basic auditory cognitive tests. Speech in noise perception is the most important human auditory capacity and a consistent problem for persons with hearing disorders. Such tests may reveal the basic auditory factors that determine speech-in-noise understanding and enable more robust, language-independent clinical diagnosis. Rönnberg et al. discusses the ongoing trend of including more cognitive factors in this effort to add to the classical models based on system identification approaches to peripheral hearing mechanisms. He proposes the Ease of Language Understanding model, which models complex interactions of cognitive modules, such as the different memory systems, lexical access, and predictive and postdictive processes. Such models help to understand the perceptual consequences of hearing disorders and mirror the trend to include cognitive factors in hearing aids and rehabilitation.

## Author contributions

All authors listed have made a substantial, direct, and intellectual contribution to the work and approved it for publication.

## References

[B1] Belin P. Zatorre R. J. Lafaille P. Ahad P. Pike B. (2000). Voice-selective areas in human auditory cortex. Nature 403, 309–312 10.1038/35002078 10659849

[B2] Gauthier I. Tarr M. J. Anderson A. W. Skudlarski P. Gore J. C. (1999). Activation of the middle fusiform ‘face area' increases with expertise in recognizing novel objects. Nat. Neurosci. 2, 568–573 10.1038/9224 10448223

[B3] Goodale M. A. Milner A. D. (1992). Separate visual pathways for perception and action. Trends Neurosci. 15, 20–25. 10.1016/0166-2236(92)90344-8 1374953

[B4] Griffiths T. D. Büchel C. Frackowiak R. S. Patterson R. D. (1998). Analysis of temporal structure in sound by the human brain. Nat. Neurosci. 1, 422–427 10.1038/1637 10196534

[B5] Gutschalk A. Mase R. Roth R. Ille N. Rupp A. Hähnel S.. (1999). Deconvolution of 40 Hz steady-state fields reveals two overlapping source activities of the human auditory cortex. Clin. Neurophysiol. 110, 856–868 10.1016/S1388-2457(99)00019-X 10400199

[B6] Lütkenhöner B. Krumbholz K. Lammertmann C. Seither-Preisler A. Steinsträter O. Patterson R. D. (2003). Localization of primary auditory cortex in humans by magnetoencephalography. Neuroimage 18, 58–66 10.1006/nimg.2002.1325 12507443

[B7] Mishkin M. Ungerleider L. G. (1982). Contribution of striate inputs to the visuospatial functions of parieto-preoccipital cortex in monkeys. Behav. Brain Res. 6, 57–77. 10.1016/0166-4328(82)90081-X 7126325

[B8] Oxenham A. J. Bernstein J. G. Penagos H. (2004). Correct tonotopic representation is necessary for complex pitch perception. Proc. Natl. Acad. Sci. U. S. A. 101, 1421–1425 10.1073/pnas.0306958101 14718671 PMC337068

[B9] Rauschecker J. P. Tian B. (2000). Mechanisms and streams for processing of “what” and “where” in auditory cortex. Proc. Natl. Acad. Sci. U. S. A. 97, 11800–11806. 10.1073/pnas.97.22.11800 11050212 PMC34352

[B10] Schneider G. E. (1969). Two visual systems. Science 163, 895–902 10.1126/science.163.3870.895 5763873

[B11] Sigalovsky I. S. Fischl B. Melcher J. R. (2006). Mapping an intrinsic MR property of gray matter in auditory cortex of living humans: a possible marker for primary cortex and hemispheric differences. Neuroimage 32, 1524–1537 10.1016/j.neuroimage.2006.05.023 16806989 PMC1839042

[B12] Strutt J. W. (1907). On our perception of sound direction. Philos. Mag. 13, 214–232.

